# The Relationship between Family Factors and Academic Achievement of Junior High School Students in Rural China: Mediation Effect of Parental Involvement

**DOI:** 10.3390/bs14030221

**Published:** 2024-03-08

**Authors:** Xiaoxia Gu, Norlizah Che Hassan, Tajularipin Sulaiman

**Affiliations:** Faculty of Educational Studies, Universiti Putra Malaysia, Serdang 43400, Selangor, Malaysia; xiaoxiagu328@gmail.com (X.G.); tajulas@upm.edu.my (T.S.)

**Keywords:** socioeconomic status, family environment, parental involvement, academic achievement

## Abstract

This study aims to understand how socioeconomic status and the family environment impact students’ academic achievement through the mediation of parental involvement in rural China. To achieve this, a cross-sectional design was adopted, and a total of 525 parents of rural junior high school students from S province in southwest China were surveyed. The proposed conceptual framework was tested by structural equation modeling. The results claimed that both socioeconomic status and the family environment are important factors affecting the academic achievement of rural students, and the role of the family environment is more pronounced. Furthermore, parental involvement has a significant mediating effect between socioeconomic status and academic achievement, especially between the family environment and academic achievement. The findings highlighted the importance of the family environment and parental involvement to compensate for the negative impact of disadvantaged family socioeconomic status on academic achievement.

## 1. Introduction

Education is a symbol of social progress and the cornerstone of national development. The performance of children in the basic education stage not only affects their achievement in the future but also the quality of a nation’s labor force [1]. In China, basic education consists of the primary and junior high school stages. Children in junior high school are at the beginning of puberty, which is a critical period for individual growth and even more so for academic and promotional competition [2]. The college entrance examination in China is the main way to enter college, and academic performance during junior high school is directly related to the opportunities for high school and even university advancement. However, the issue of educational inequality in China has always been evident [3], with rural education often at a disadvantage [4,5], a trend that continues at the junior high school stage.

It is an indisputable fact that the academic achievement of rural students is lower than that of urban students, which has been verified in different countries [6,7,8]. In China, the reasons for this discrepancy are twofold. One is the impact of macro-level institutional arrangements, such as China’s urban-rural divide according to the household registration system [4,9]. The second is the influence of the micro-level of the family, such as the family’s opportunity and ability to participate in education [6,10,11,12,13]. Therefore, it is important to pay special attention to the family dimension when analyzing differential outcomes in academic achievement. In China, family education has also attracted attention at the national level. On 23 October 2021, the Family Education Promotion Law of the People’s Republic of China was promulgated and officially implemented in 2022. It legally defines the responsibilities and obligations of families and parents in their children’s education.

The family has a profound effect on academic achievement. Bronfenbrenner argued that microsystems can have a lasting impact on individuals for a long period of time [14]. In the family, individuals can develop cognitive and physical skills and accumulate rich experiences. Numerous previous studies have confirmed that family socioeconomic status is a significant predictor of academic achievement. A higher family socioeconomic status indicates high academic achievement, while low socioeconomic status is considered an important environmental determinant of low academic achievement [15,16,17,18]. In addition, a good family environment also has a positive impact on academic achievement [19,20,21], which is a reflection of a soft family atmosphere. Children in families with high cohesion [22,23,24], low tensions and conflict [25,26,27], and emphasis on cultural value [20,28,29] have better academic achievement. Even if children in high-risk family conditions, such as rural areas with low socioeconomic status, have lower overall achievement, the family is still oriented toward shaping the development of children who have the potential for academic success. Recent studies found that parents of low family socioeconomic status may be more inclined to use adaptive strategies, such as counteracting the resulting potential risks by strengthening the soft home environment and increasing parental involvement [10,30,31], to help create favorable conditions for their children’s academic success. However, research on protective factors to alleviate the adverse effects of family socioeconomic status on academic achievement is still limited among Chinese students.

For students in middle school, the differences in the importance of various family factors on academic achievement, and the mechanisms of these influences are unclear. Some evidence has consistently identified parental involvement as an important factor mediating the relationship between family socioeconomic status and academic achievement [11,16,32,33]. The role of parents in influencing the educational behaviors and academic achievement of children from lower socioeconomic backgrounds has been increasingly emphasized [30,34], particularly accentuated by the impact of the 2019 COVID-19 pandemic [13,35]. However, few studies have considered the comprehensive impact of both family socioeconomic status and the family environment on academic achievement, particularly in rural settings. Additionally, the nuanced role of parental involvement in this context has been understudied. Therefore, this study aims to delve into the multidimensional family factors contributing to academic achievement among rural junior high school students and further elucidate the underlying mechanisms. Specifically, our investigation will scrutinize and compare the pivotal roles played by family socioeconomic status, familial environment, and parental involvement in shaping the academic achievement of students. An analysis of family education focusing on class perspectives is useful in clarifying how a family’s class-cultural strengths or weaknesses affect the academic achievement of its offspring.

## 2. Literature Review

### 2.1. Socioeconomic Status and Academic Achievement

Socioeconomic status is a reflection of the objective background of the family. Currently, research on the impact of family factors on children’s academic achievement is mostly analyzed from the perspective of family socioeconomic status, such as parents’ education level, occupation, and family income [6,36]. Evidence from various regions indicates a positive, weak to moderate correlation between family socioeconomic status and academic achievement [18,37,38,39]. Moreover, another study shows that family socioeconomic status is considered to be the most significant factor affecting academic achievement, especially during the children’s minor years [40].

In China, the association between socioeconomic status and academic achievement is more pronounced than it is in some developed and developing countries. A meta-analysis by Liu et al. of family socioeconomic status and academic achievement in China indicated that the relationship is stronger than in the USA [41], which is also above the extent of association in developing countries in general [42]. The role of family socioeconomic status in individual education acquisition has not weakened with the expansion of education enrollment in China but rather shows an upward trend [33]. However, some studies have pointed out that the relationship between family socioeconomic status and academic achievement is generally on a decreasing trend [18,41]. In this way, findings on the differential effect of family socioeconomic status on academic achievement suggest that the exact effects and the mechanisms involved remain to be further explored.

### 2.2. Family Environment and Academic Achievement

The family environment discussed in this study is viewed as a means of evaluating family relationships and the overall atmosphere, emphasizing the soft environmental factors contained within it. Characteristics of the family environment have been found to be associated with students’ academic achievement [19,20,21]. A warm family atmosphere allows children to focus on their development. On the one hand, positive family environments, such as family cohesion [24], family support, and home academic culture [43,44], are considered protective factors in favor of academic achievement. On the other hand, negative family environments, such as a high degree of family conflict, are a risk factor for poor academic achievement [25,27].

In addition, the family environment is particularly important for families in disadvantaged classes, such as those with low socioeconomic status. Parents in these families have difficulty transmitting the cultural rules of the advantaged class to their children [45]. However, a study by Yamamoto et al. emphasized the strengths of low socioeconomic status families, where parents exhibited a less stressful family environment and stronger beliefs about parental responsibility for education [46]. Hence, parents of low socioeconomic status can also maximize their children’s lives and academic development if they focus on their children’s education by adopting an “active cultivation” model of parenting and creating a home environment that positively and effectively supports their children’s well-being and academic achievement [47,48,49].

### 2.3. Parental Involvement and as a Mediator

Parental involvement is the process by which parents are involved in their children’s academic life in a variety of ways, such as academic guidance, behavioral supervision, and parent-child interaction, to promote their children’s education and development to the greatest extent possible [50,51,52]. In general, parental involvement includes two types: school-based involvement and home-based involvement [53,54]. School-based involvement focuses primarily on the process of parental involvement at school, such as attending school events and interacting with teachers, whereas home-based involvement emphasizes parents’ educational involvement activities at home, such as home supervision and academic guidance [54].

Numerous researchers have noted that parental involvement is critical to children’s academic success and can positively predict their academic achievement, such as higher grades and greater engagement [51,52,55,56,57,58]. In this study, we focus on home-based involvement, as in the cultural context of China and even all of Asia, parents are more involved in home-based activities, which are more important for their children’s academic achievement [39,54,59]. Moreover, home-based involvement is also considered to play an increasingly important role in the middle school stage [60,61].

Considering the different resources and knowledge that families bring to children’s education, it seems reasonable to expect that parental involvement in education varies systematically depending on family factors. There is a large body of evidence that has emphasized the mediating role of parental involvement in the mechanism by which family socioeconomic status affects academic achievement [10,11,33,34]. Family socioeconomic status affects children’s academic achievement by influencing the level of parental involvement in education. A high level of family socioeconomic status increases parental involvement in their child’s development [58], while parents with lower income and lower education are less likely to be involved in their child’s education, either at home or at school [62,63,64].

On the other hand, however, increasing parental involvement is an effective way to close the achievement gap for families of low socioeconomic status. That is, for disadvantaged children, parental involvement can also compensate for their relative lack of family background resources and narrow the gaps in cognitive ability and cultural capital with other students from relatively advantaged backgrounds [30,34]. According to Cooper et al., families in greater poverty have a higher level of parental intervention than autonomy support [31]. Meanwhile, parental involvement is also a mediating variable in the influence of the family environment on academic achievement. Based on Unger et al., family support, such as family cohesion and parental involvement, can partially mediate the adverse effects of parental conflict on adolescent academic achievement [26]. As a result, parents in disadvantaged families also actively seek alternative ways to participate in their children’s education to mitigate the negative impact of unfavorable family conditions on their children’s development.

### 2.4. The Context of This Study

Educational systems and perceptions vary across societies and cultures, which directly affects children’s academic outcomes. Due to the dual urban-rural division system in China, education is also divided into two completely different states, namely rural and urban, and rural children are relatively disadvantaged in terms of academic achievement [4,5,65]. It is often difficult to change institutional inequalities, but the role of the family, as the key micro-system influencing children’s schooling and development, is crucial. For rural families, the impact of socioeconomic status [6,18], the family environment [10,30], and parental roles [34] on children’s academic achievement is likely to be different from that of urban families. However, there has been insufficient research addressing how different family factors are related to the academic achievement of disadvantaged students (e.g., low socioeconomic status backgrounds) in China through parental involvement.

**Figure 1:**
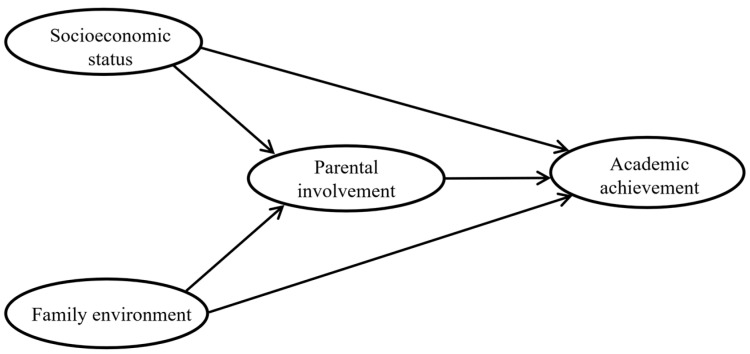
Structural model hypothesized for this study.

To address this, we hypothesized that family socioeconomic status, the family environment, and parental involvement are significant predictors of academic achievement among rural junior high school students in China. Socioeconomic status and the family environment may motivate parents to become more involved in their children’s education, which in turn could predict an improvement in students’ academic achievement. In particular, it emphasizes the unique role of the family environment. Therefore, this study proposed the hypothetical model in Figure 1 below to present the associations between the above constructs, and structural equation modeling was used to test these relationships. In total, the following three questions are addressed in this hypothetical model:

(1) To what extent can socioeconomic status and the family environment predict the academic achievement of rural junior high school students in China?
(2) Does the family environment have a more significant impact on the academic achievement of rural junior high school students in China, to some extent compensating for the adverse effects of their disadvantaged socioeconomic status?
(3) To what extent does parental involvement mediate the relationship between socioeconomic status and academic achievement, as well as the relationship between the family environment and academic achievement?

## 3. Materials and Methods

### 3.1. Participants

A total of 525 parents of rural junior high school students in S Province, China, were selected as participants. According to the demographic results, 58.86% (*n* = 309) of the participants were mothers, and 41.14% (*n* = 216) were fathers. Regarding the students’ gender, 50.67% were boys (*n* = 266), and 49.33% were girls (*n* = 259). It encompasses students from various grades of junior high school, with 37.14% from Grade 7 (*n* = 195), 41.52% from Grade 8 (*n* = 218), and 21.33% from Grade 9 (*n* = 112), respectively. It was determined that a sample size of 138 was necessary after power analysis (effect size = 0.15, power = 0.95, alpha = 0.05) was carried out using G*POWER 3.1 [66]. This indicates that there were far more valid participants overall in this study than the minimal number recommended above.

This study used purposive sampling as it aimed to recruit participants with the following characteristics: low socioeconomic status families from rural areas with children attending rural schools. Considering the requirements of socioeconomic status, families with rural registered residence but their children studying in cities were excluded from this study. After the eligible participants were identified, a random selection process was employed to determine respondents for questionnaire completion. 550 participants filled out the questionnaire in this study, and 25 participants were dropped due to providing incorrect data. Thus, the final sample size was 525, with a return rate of 95.45%. S Province is located in the southwest of China. Due to the uneven development of China’s eastern, central, and western regions, education in the rural areas of the west is significantly lagging. Thus, S Province serves as a representative example, highlighting the remarkable educational disparities in rural areas.

### 3.2. Measures

#### 3.2.1. Socioeconomic Status

According to previously accepted recommendations [36], the three main indicators of family socioeconomic status are education, occupation, and income. Socioeconomic status in this study was also measured by five items of these three dimensions, including the father’s education level, the mother’s education level, the father’s occupation, the mother’s occupation, and the family’s monthly income (see Appendix A). All items on socioeconomic status were rated on a 5-point scale ranging from 1 to 5. First, the educational level of both parents was assessed as: 1 = Elementary school and below; 2 = Junior high school degree; 3 = Senior high school degree; 4 = Junior college degree; 5 = Bachelor’s degree and above. Second, the parents’ occupations were assessed as: 1 = Unemployed; 2 = Farmers; 3 = Production and Manufacturing Workers; 4 = Technical and Skilled Workers; 5 = Professional and Managerial Roles. Third, the family monthly income was assessed as: 1 = RMB2000 and below; 2 = RMB2000–RMB4000; 3 = RMB4000–RMB6000; 4 = RMB6000–RMB8000; 5 = RMB8000 and above. 

#### 3.2.2. Family Environment

The Chinese version of the Family Environment Scale (FES-CV), which was revised by Phillips based on the Family Environment Scale (FES) [67], was adopted to measure the family environment of participants. The original FES was developed by Moos and Moos to measure the family environment in Western countries [68], and Phillips adjusted the scale to form a Chinese version based on the cultural background of China. This study selected four subscales of the FES-CV that have satisfactory validity and internal consistency and are also suitable for the Chinese cultural context (see Appendix A). Specifically, they are cohesion (e.g., “Our family members always give each other the utmost help and support”), conflict (e.g., “There are frequent quarrels at home”), intellectual–cultural orientation (e.g., “Our family often talks about politics and social issues”), and organization (e.g., “Larger activities in the home are carefully planned”). These four subscales contain 36 items, with each subscale containing 9 items, and all items are scored as “yes” and “no”. The score of each subscale was calculated by adding up the nine items, with a range of 0–9. The higher the score, the better the family atmosphere, except for conflict. Conflict represents the inverse dimension in the original scale, where a lower score signifies a better family environment. Therefore, we reversed the scoring of all items in this subscale to maintain consistency with the direction of the other three dimensions. All four subscales have an acceptable Cronbach’s alpha ranging from 0.63 to 0.75 (cohesion = 0.75, conflict = 0.67, intellectual–cultural orientation = 0.64, and organization = 0.63).

#### 3.2.3. Parental Involvement

From a cultural capital perspective, parental involvement is considered to be a process in which parents are involved in transmitting cultural capital through home education [45]. Based on this theory, Ho’s home-based parental involvement scale was adopted and adjusted to form the parental involvement scale for this study [54]. The original scale consisted of 15 items in 4 dimensions, including learning support (5 items), home enrichment (4 items), home supervision (4 items), and home limitation (2 items). The overall reliability of the scale was 0.843, which is satisfactory.

Two aspects of the parental involvement scale for this study are noteworthy. First, the original scale was designed for students. However, considering that the participants in this study were parents, some of the items were adjusted to be more consistent with parental expressions. For example, the original item, “Check your homework” was modified to “Check your child’s homework”. Second, with the changing times, electronic devices, such as mobile phones, have gradually become an important factor affecting adolescents’ academic performance, and overuse and internet addiction cause a decline in academic achievement [69,70,71]. Thus, to better reflect parents’ restrictions on their children’s entertainment, a new item was added to this study, which was “Restrict time of playing with electronic devices (e.g., computer, smartphone)”. The final version of the parental involvement scale expanded the original 15 items to 16 (see Appendix A). It was reconfirmed as three factors for the final analysis, home enrichment (6 items), home supervision (6 items), and home restrictions (3 items), respectively (see the exploratory factor analysis and confirmatory factor analysis results below). The above behaviors were rated on a 4-point scale, namely 1 = Never, 2 = Seldom, 3 = Sometimes, 4 = Often.

#### 3.2.4. Academic Achievement

The subjective scoring method, a self-reported assessment of academic achievement, is used to measure students’ academic achievement [72,73]. Since there are different subjects in each grade and inconsistent evaluation criteria for exam scores at junior high schools in China, it is appropriate to use the subjective scoring method to measure academic achievement in this study. Chinese, math, and English are the three main subjects in China [74], and this is also true at the junior high school stage. Therefore, the academic achievement of this study was evaluated with four items, namely Chinese, math, English, and overall achievement level (see Appendix A). Responses were obtained along a 4-point scale, namely 1 = Poor, 2 = Average, 3 = Good, and 4 = Excellent.

### 3.3. Procedure

Prior to the commencement of data collection, ethical approval was obtained from the Ethics Committee of the University Putra Malaysia (JKEUPM) as this study involves human subjects. Participants were also assured that the information was confidential and anonymous. Subsequently, informed consent to participate in this study was obtained from all the parents and schoolteachers. At the beginning of data collection, the researchers contacted the teachers who were selected and agreed to participate in the survey and entrusted them to use the Questionnaire Star tool to send electronic questionnaires to the parents. The form link was shared by teachers on WeChat. It has been ensured that the questionnaire was distributed to at least 4 different classes in each school, covering a total of 6 schools. Meanwhile, during the distribution of the questionnaire, an introduction letter was attached to explain the purpose and content of the study and to emphasize that participants had the right to refuse to fill in the answers. The questionnaire took about 20 min, and it was completed by only one parent of the student (either the father or the mother). The purpose of this measure was to prevent duplicate data for the same student in the dataset. Additionally, participants are allowed to complete the questionnaire independently or in consultation with their spouses.

### 3.4. Data Analysis

Quantitative data were analyzed using two software packages, namely SPSS version 26.0 and AMOS version 24.0 [75]. The analysis procedures included exploratory factor analysis (EFA), a test for common method bias (CMB), confirmatory factor analysis (CFA), correlation analysis, and structural equation modeling (SEM). First, 127 parents were selected as participants in a pilot study, which was a separate dataset from the formal data. To clarify the factors of the revised parental involvement scale in this study, the pilot data was analyzed by performing the EFA in SPSS. In the subsequent CFA, this outcome was confirmed once more. Second, after performing CMB, we conducted CFA for each construct, including socioeconomic status (SES), family environment (FES), parental involvement, namely home enrichment (HE), home supervision (HS), home restrictions (HR), and academic achievement (AA), to test how well indicators measure individual constructs [76]. The maximum likelihood method was utilized for estimation. Third, correlation analysis among all constructs was employed. Finally, to test the hypothesized model, the structural equation modeling (SEM) technique was employed using AMOS, which consists of two parts, the measurement model and the structural model [77]. The measurement model defines the relationship between observed and latent variables, and the structural model tests the hypothesized relationships. In addition, factor loadings, average variance explained (AVE), composite reliability (CR), and Cronbach’s alpha were used to check the validity and reliability of each construct. Next, multiple benchmarks, such as the ratio of χ^2^/df, comparative fit index (CFI), normal fit index (NFI), goodness-of-fit index (GFI), Tucker-Lewis Index (TLI), and root mean square error of approximation (RMSEA) were used to evaluate the model fit. To test the mediation effect of parental involvement, the bootstrap method with 5000 times resampling (95% percentile confidence level) was applied [78].

## 4. Results

### 4.1. Exploratory Factor Analysis

The EFA was conducted to reclassify the factors of the items in the parental involvement scale. The results are shown in Table 1. It indicates that the sample of the pilot was suitable for factor analysis, as KMO = 0.908, Sig. (Bartlett’s Test) = 0.000 < 0.05. The initial 16 items were retained and grouped into three factors: home enrichment (6 items), home supervision (7 items), and home restrictions (3 items). The total variance explained by these three factors was 72.63% > 50%. The Cronbach’s α for each factor ranged from 0.816 to 0.951, with a total coefficient of 0.944. Factor loadings for all items ranged from 0.491 to 0.847, which were all above 0.40 [76].

### 4.2. Common Method Bias

Since the data in this study were all self-reported by the participants, it was necessary to conduct a common method bias test for the variables used. Harmon’s single-factor test was performed by including all items in the questionnaire [79]. The results showed that there were six factors with eigenvalues greater than 1, which together explained 68.928% of the total variance. The first factor explained 34.329%, which was less than the criterion of 40%. Therefore, it can be considered that there was no obvious problem of common method bias.

### 4.3. Validity and Reliability 

The CFA was carried out separately for each latent variable to prepare for SEM analysis. Table 2 shows the evaluation results of fit indices for all constructs. Convergent validity refers to a set of indicators that presume to measure a construct [80], and two methods can be used to test it, namely factor loadings and average variance extracted (AVE). The factor loading on a factor higher than 0.5 indicates that it has high convergent reliability [76,77]. In this study, the factor loading values for each construct were greater than 0.5, excluding HS4, which was removed due to its low factor loading. A high AVE value also indicates high convergent validity and is required to be greater than 0.5 [76,81]. According to Table 2, the AVEs for SES, HE, HS, HR, and AA ranged from 0.513 to 0.638, all of which are greater than 0.5. Although the AVE value of FES is 0.442, slightly below 0.5, it is still above the acceptable level of 0.4 [81]. Next, the composite reliability (CR) of each construct was checked in the study. If the CR is greater than 0.7, it indicates the instrument is considered reliable [76]. The results indicate that all constructs have a CR range from 0.755 to 0.914, which is above the criterion of 0.7. At the same time, Cronbach’s α for all constructs ranges from 0.759 to 0.919, indicating good reliability. Thus, the validity and reliability of all scales were proven.

### 4.4. Test of the Measurement Model

After assessing each construct, all latent variables were included in a final model for testing. The results of the measurement model have shown a good level of fit: χ^2^/df = 2.688, CFI = 0.934, NFI = 0.900, GFI = 0.881, TLI = 0.926, RMSEA = 0.057. This means that the observation data fits well [82]. 

### 4.5. Correlation Analysis

Table 3 presents the Pearson correlation results between key variables, including socioeconomic status, the family environment, parental involvement (home enrichment, home supervision, and home restrictions), and academic achievement. It can be seen that all variables are significantly correlated with each other. Specifically, socioeconomic status is positively associated with the family environment (r = 0.211), parental involvement (r = 0.269), and academic achievement (r = 0.247). The family environment is positively related to parental involvement (r = 0.589), and academic achievement (r = 0.296). Additionally, parental involvement is positively correlated with academic achievement (r = 0.372). There is a relatively high degree of correlation between various variables, which provides support for further testing of the hypothetical relationships.

### 4.6. Test of the Structural Model 

Based on the above measurement model and correlation results, structural equation modeling (SEM) was adopted to test the relevant assumptions by performing a path analysis. Among them, socioeconomic status and the family environment were independent variables, academic achievement was the dependent variable, and parental involvement was the mediating variable. Following the process of analyzing the mediating effect [83], the main effect of socioeconomic status and the family environment on academic achievement was first discussed. Subsequently, parental involvement was added as a mediating variable in the final structural model.

### 4.7. Direct Effect 

**Figure 2:**
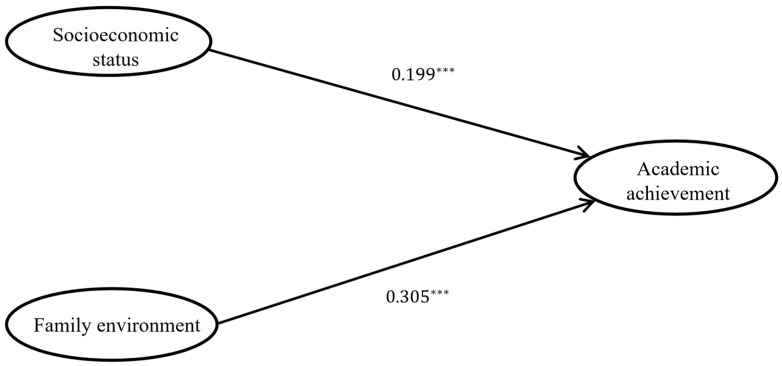
Main effect of SES and FES on AA. *** *p* < 0.001.

At first, a structural model of socioeconomic status and the family environment on academic achievement in the absence of parental involvement was constructed (as shown in Figure 2). The model fitness index results are χ^2^/df = 1.711, CFI = 0.987, NFI = 0.969, GFI = 0.973, TLI = 0.982, and RMSEA = 0.037, which indicates that the model has a good fitness for the data. According to Figure 2, the path coefficients from socioeconomic status to academic achievement, as well as from the family environment to academic achievement, are statistically significant with *p* values less than 0.001. In particular, the family environment has a stronger effect on academic achievement (β = 0.305) than socioeconomic status on academic achievement (β = 0.199).

### 4.8. Mediation Effect

**Figure 3:**
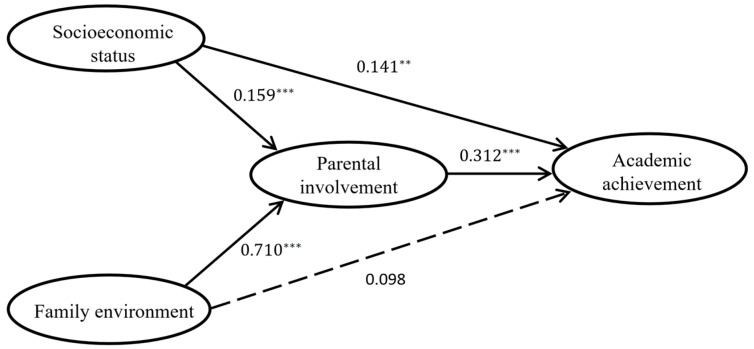
Mediation model. ** *p* < 0.05; *** *p* < 0.001.

In order to determine whether parental involvement mediates the relationship between socioeconomic status and academic achievement, as well as the relationship between the family environment and academic achievement, this research constructed the mediation model using AMOS (as shown in Figure 3). The three dimensions of parental involvement were included in one second-order factor. The overall fit statistics of the model indicate a good fit: χ^2^/df = 2.625, CFI = 0.937, NFI = 0.902, GFI = 0.884, TLI = 0.929, RMSEA = 0.056. 

As shown in Figure 3, in the path of socioeconomic status → parental involvement → academic achievement, socioeconomic status has a significant positive effect on parental involvement (β = 0.159, *p* = 0.000), and parental involvement has a significant positive effect on academic achievement (β = 0.312, *p* = 0.000). Meanwhile, socioeconomic status also has a positive impact on academic achievement, and after adding parental involvement as a mediating variable, the path coefficient decreased from 0.199 to 0.141, but it is still significant (*p* = 0.003). It suggests that parental involvement partially mediates the relationship between socioeconomic status and academic achievement. In terms of the path of family environment → parental involvement → academic achievement, the family environment has a positive and significant impact on parental involvement (β = 0.710, *p* = 0.000). Similarly, parental involvement has a positive and significant impact on academic achievement (β = 0.312, *p* = 0.000). It can be seen that after adding parental involvement as the mediation variable, the path coefficient of the family environment on academic achievement decreased from 0.305 to 0.098 and became insignificant (*p* = 0.281). This indicates that parental involvement mediates the effect of the family environment on academic achievement and serves as a full mediator.

Then, a bootstrap method with a sample size of 5000 was performed to test the stability of this mediation model. Meanwhile, the proportion of mediating effects of parental involvement in different pathways was further calculated. Table 4 shows the results of the mediating effect size of parental involvement. It can be seen that the total indirect effect of the model is 53.137%. This indicates that the mediation effect of parental involvement accounts for 53.137% of the total effect in explaining the relationship between the independent variables (socioeconomic status and the family environment) and the dependent variable (academic achievement). The total indirect effect consists of two parts: the first is the path of indirect effect of parental involvement between socioeconomic status and academic achievement, and the second is the path of indirect effect of parental involvement between the family environment and academic achievement. Furthermore, in Path 1, the indirect effect of parental involvement was significant (standardized indirect effect β = 0.050, *p* = 0.002, 95% CI range 0.013 to 0.095) and accounted for 9.804% of the total effect. In path 2, the indirect effect of parental involvement was also significant (standardized indirect effect β = 0.221, *p* = 0.002, 95% CI range from 0.053 to 0.256), accounting for 43.333% of the total effect. It shows that the mediating role of parental involvement between the family environment and academic achievement is greater than that between socioeconomic status and academic achievement.

## 5. Discussion

In the present study, we aimed to understand the impact of family factors on parental involvement in facilitating the academic achievement of junior high school students in rural China. This study investigated the family socioeconomic status, the family environment, parental involvement, and academic achievement of students in China. With the SEM analysis and a meditation test, the relationships among these constructs were examined. The following are some of the main findings based on the Chinese context.

### 5.1. The Roles of Socioeconomic Status and the Family Environment in Predicting Academic Achievement

This study takes into account both family socioeconomic status and the family environment when considering family factors, and the results show that both of them are correlated with the academic achievement of rural junior high school students in China. In other words, family factors have a comprehensive impact on academic achievement. Family socioeconomic status represents the objective condition of the family, and a large number of previous studies identified the facilitating role of socioeconomic status in students’ academic achievement [15,16,18,42]. A favorable family socioeconomic status, such as higher family income, better education, and occupation of the parents, is conducive to improving the academic achievement of the child [11]. For rural junior high school students in China, the overall socioeconomic status of the family is lower than the average level, but its impact on children’s academic achievement is still significant. This indicates that the role of family socioeconomic status remains important among students from disadvantaged social groups.

The examination of the family environment in this study, on the other hand, was designed to verify the effect of the soft family atmosphere on students’ academic achievement. The results of the analysis in terms of family cohesion, conflict, intellectual–cultural orientation, and organization reveal that a good family environment has a significant positive impact on students’ academic achievement. That is, families with stronger cohesion, cultural identity, and organization and fewer conflicts are associated with better academic achievement among children. This is similar to some of the findings of previous studies. According to Ghazarian and Buehler, interparental conflict was a risk factor for lower academic achievement among youth, reducing their potential to excel in academic endeavors [27]. Besides, some evidence has suggested that the family atmosphere may serve as a pathway to compensate for the adverse effects on academic achievement of disadvantaged students due to inadequate socioeconomic conditions in their families [10,13,30]. The results of the structural model constructed to analyze the academic achievement of the rural junior high school students in China in this study further validate the previous findings. According to the main model of academic achievement without a mediator, the family environment plays a greater role than socioeconomic status. This finding further emphasizes the special importance of a soft family environment for the academic achievement of students from disadvantaged backgrounds, such as rural areas.

### 5.2. The Mediating Role of Parental Involvement

Our study delves into the evidence of the pathway (parental involvement as a mediator) by which socioeconomic status, embodied as the objective conditions of the family and the family environment, embodied as the soft atmosphere of the family, influence academic achievement, which greatly further enriches the understanding of the mechanism of family factors on academic achievement. At first, the results of the mediation test in this study revealed that the effect of socioeconomic status on academic achievement is partially mediated by parental involvement in rural China. This is in line with previous studies, which found that parental involvement is an important mediator. As Davis–Kean highlighted, socioeconomic status affects children’s academic achievement to a large extent through parental beliefs and behaviors [84]. Parents with good family socioeconomic status are more likely to increase their involvement in their children’s education, such as by providing abundant learning resources and monitoring learning, which in turn enhances their children’s academic achievement [16,33,34,64]. Parental involvement is also an effective way to close the achievement gap for families with low socioeconomic status [30,64]. The results of this mediation analysis re-emphasize the importance of family socioeconomic status on academic achievement, even for families from disadvantaged groups.

Furthermore, it is worth noting that parental involvement has the strongest mediating effect between the family environment and academic achievement, which is the most significant contribution of the study. That is, the family environment has a significant effect on academic achievement by increasing the level of parental involvement. This finding may be derived from the fact that the family has a subtle and continuous influence on children’s development [85]. Therefore, from an intra-family perspective, we emphasize the critical importance of parents in this process. They not only enrich the learning environment and resources but also play a key role in creating a positive and conducive atmosphere for children’s learning. The better the family environment (e.g., high cohesion and cultural orientation, low conflict, etc.), the more parents are actively involved in their children’s academics to provide them with support and supervision, which in turn enhances academic achievement. Meanwhile, this study specifically emphasizes the important impact of parental involvement on children from underprivileged backgrounds. It has a crucial function in compensating for the impact of unfavorable family environments (e.g., inter-parental conflict) on academic achievement [26,86,87]. Therefore, it is argued that by upgrading the level of parental educational involvement, the family environment can be a useful means of helping children from disadvantaged backgrounds to minimize the impact of poor socioeconomic conditions and thereby enhance their academic achievement.

### 5.3. Limitations and Future Research

There are some limitations to this study that are noteworthy. First, our study only examined home-based involvement and did not include other components, such as school-based involvement. Future research should include a more comprehensive measure of parental involvement in the analysis. Similarly, the measure of academic achievement used in this study was a subjective assessment. Although such a measure reported by students or others (e.g., teachers, parents, etc.) has been frequently used before [85], future related studies should adopt objective standardized measures (e.g., test scores) to measure academic achievement. Second, this study is a cross-sectional design, and it can objectively reflect the relationship between the variables, but it is unable to provide a longitudinal and developmental perspective. Therefore, future research should consider a longitudinal design to better understand the process by which family factors, particularly the family environment and parental roles, contribute to children’s academic achievement. Third, only rural junior high school students in S Province were selected for this study. On the one hand, with China’s large population and significant regional differences in rural areas, the participants in S province may have limited the collection of a more representative sample. On the other hand, this study focused only on the junior high school level, which is hardly equivalent to the situation at different stages of education. Thus, there is a need for future research to consider a larger scope and more school-level groups to conduct the study.

## 6. Conclusions

As an empirical exploration of family factors influencing the academic achievement of rural junior high school students in a Chinese cultural context, this study enriches the theme that the family and parents are responsible for their children’s academic outcomes. We found that a good socioeconomic status and the family environment are associated with better academic achievement. Parental involvement plays a mediating role in the relationship between family socioeconomic status and academic achievement and in the relationship between the family environment and academic achievement. Moreover, for rural junior high school students, the family environment and its role in academic achievement through parental involvement are more important than socioeconomic status. Thus, a positive family atmosphere plays a significant compensatory role for rural students.

## Figures and Tables

**Table 1:** Results of exploratory factor analysis for parental involvement.

Factors	Items	Component (Rotated Factor Loading)		
		1	2	3
Home enrichment (HE) α = 0.951	HE1	0.847		
	HE2	0.831		
	HE3	0.844		
	HE4	0.829		
	HE5	0.831		
	HE6	0.774		
Home supervision (HS) α = 0.900	HS1		0.491	
	HS2		0.688	
	HS3		0.641	
	HS4		0.751	
	HS5		0.725	
	HS6		0.752	
	HS7		0.637	
Home restrictions (HR) α = 0.816	HR1			0.822
	HR2			0.809
	HR3			0.776
Kaiser-Meyer-Olkin Measure of Sampling Adequacy (KMO)		0.908		
Sig. of Bartlett’s Test of Sphericity		0.000		
% variance explained		72.630%		

**Table 2:** Validity and reliability of the constructs.

Latent Variable	No	Item	Standardized Factor Loading	Cronbach’s Alpha	CR	AVE
Socioeconomic status (SES)	5	SES1	0.620	0.859	0.839	0.513
		SES2	0.700			
		SES3	0.750			
		SES4	0.840			
		SES5	0.650			
Family environment scale (FES)	36	FES1	0.771	0.759	0.755	0.442
		FES2	0.591			
		FES3	0.503			
		FES4	0.756			
Home enrichment (HE)	6	HE1	0.780	0.919	0.914	0.638
		HE2	0.750			
		HE3	0.820			
		HE4	0.790			
		HE5	0.820			
		HE6	0.830			
Home supervision (HS)	6	HS1	0.570	0.887	0.879	0.553
		HS2	0.790			
		HS3	0.590			
		HS5	0.870			
		HS6	0.830			
		HS7	0.760			
Home restrictions (HR)	3	HR1	0.930	0.806	0.823	0.617
		HE2	0.820			
		HR3	0.560			
Academic achievement (AA)	4	AA1	0.770	0.878	0.873	0.633
		AA2	0.820			
		AA3	0.750			
		AA4	0.840			

**Table 3:** Correlation coefficient matrix of key variables.

Construct	1	2	3	4	5	6	7
1. Socioeconomic status	-						
2. Family environment scale	0.211 ***	-					
3. Home enrichment	0.265 ***	0.543 ***	-				
4. Home supervision	0.237 ***	0.549 ***	0.724 ***	-			
5. Home restrictions	0.131 **	0.311 ***	0.302 ***	0.490 ***	-		
6. Overall parental involvement	0.269 ***	0.589 ***	0.897 ***	0.920 ***	0.602 ***	-	
7. Academic achievement	0.247 ***	0.296 ***	0.374 ***	0.356 ***	0.114 **	0.372 ***	-

** *p* < 0.05; *** *p* < 0.001.

**Table 4:** Mediation effect size and proportion.

Path	Estimate	Beta	*p* Value	95% LLCI	95% ULCI	Proportion
Total effect	0.356	0.510	0.000	0.261	0.458	-
SES → PIL → AA	0.045	0.050	0.002	0.013	0.095	9.804%
FES → PIL → AA	0.127	0.221	0.002	0.053	0.256	43.333%
Total indirect effect	0.172	0.271	0.002	0.070	0.320	53.137%

## Data Availability

The data presented in this study are available on request from the corresponding author.

## Appendix A. Survey Items for Key Variables

**Table:**

Constructs	Survey Items
Socioeconomic Status (SES)	SES1. Education level of father. SES2. Education level of mother. SES3. Occupation of father. SES4. Occupation of mother. SES5. Your household’s total monthly income.
Family Environment Scale (FES)	
FES1. Cohesion	1. Our family members always give each other the utmost help and support. 2. We are bored at home. 3. Family members are willing to put a lot of effort into household chores. 4. In our home, there is an atmosphere of harmony. 5. ★ When something happens at home, few people do it voluntarily. 6. Family members always support each other sincerely. 7. ★ Our family has very little collective spirit. 8. Family members have always gotten along with each other. 9. Every member of the family has been given adequate attention.
FES2. Conflict	10. There are frequent quarrels at home. 11. ★ Family members rarely get angry with each other publicly. 12. Sometimes family members drop things when they get angry. 13. ★ Few tempers among family members. 14. Family members often blame and criticize each other. 15. Family members sometimes fight with each other. 16. When family members disagree, we have always avoided it to keep things amicable. 17. Family members often want to outdo each other. 18. When family members have conflicts, they sometimes quarrel loudly.
FES3. Intellectual–cultural orientation	19. Our family often talks about politics and social issues. 20. ★ We seldom go out to listen to lectures, watch plays or go to museums and exhibitions. 21. We all agree that learning something new is more important than anything else. 22. ★ We are not so interested in cultural events. 23. ★ We rarely discuss issues related to scientific and technological knowledge. 24. Someone at home plays an instrument. 25. Family members go to the library often. 26. Watching TV is more important than reading books in our house. 27. Family members enjoy music, art and literature.
FES4. Organization	28. Larger activities in the home are carefully planned. 29. Generally, we all pay attention to keeping our home in good order. 30. In our house, when something is needed, it is often not available. 31. Being on time is very important in our family. 32. People in our family often change their plans. 33. Family members take great care in keeping their rooms tidy. 34. In our family, everyone has a clear division of labor. 35. ★ Our family spends money without a plan. 36. We must have someone wash the dishes immediately after our meal.
Parental Involvement (PIL)	
Home enrichment (HE)	1. Explore more new things (e.g., go to Ocean Park or Science Museum). 2. Watch and discuss cultural TV programs with your child. 3. Bring your child along to libraries. 4. Join courses about children teaching. 5. Read books about children teaching. 6. Reading with your child.
Home supervision (HS)	1. Concern about study progress. 2. Supervise homework. 3. Provide ideal study environment for your child. 4. Help your child to pack school bag. 5. Check your child’s homework. 6. Guide your child to do homework and study. 7. Set schedules for your child.
Home restrictions (HR)	1. Restrict time of watching TV. 2. Restrict time of playing with electronic devices (e.g., computer, smartphone). 3. Restrict time of going outside.
Academic Achievement (AA)	1. Chinese. 2. Math. 3. English. 4. Overall achievement level.

★ indicate a negative meaning.
